# HIV and mental health provider experiences of implementing brief depression and suicide screening among people living with HIV in Tanzania: A qualitative study

**DOI:** 10.1371/journal.pmen.0000268

**Published:** 2025-03-13

**Authors:** Kim Madundo, Judith M. Mwobobia, Mirlene Perry, Elizabeth Knippler, Ismail Amiri, Elizabeth F. Msoka, Clotilda S. Tarimo, Victor Katiti, Blandina T. Mmbaga, David B. Goldston, Michael V. Relf, Brandon A. Knettel

**Affiliations:** 1 Department of Psychiatry and Mental Health, Kilimanjaro Christian Medical Centre, Moshi, Tanzania; 2 Department of Psychiatry and Mental Health, Kilimanjaro Christian Medical University College, Moshi, Tanzania; 3 Brown School, Washington University in St. Louis, St. Louis, Missouri, United States of America; 4 Duke University School of Nursing, Durham, North Carolina, United States of America; 5 Duke Center for AIDS Research, Durham, North Carolina, United States of America; 6 Duke Global Health Institute, Durham, North Carolina, United States of America; 7 Kilimanjaro Clinical Research Institute, Moshi, Tanzania; 8 Department of Psychiatry & Behavioral Sciences, Duke University, Durham, North Carolina, United States of America; PLOS: Public Library of Science, UNITED KINGDOM OF GREAT BRITAIN AND NORTHERN IRELAND

## Abstract

Depression and suicidal thoughts and behaviour are remarkably common among people living with HIV worldwide, leading to a higher burden of disease, poor HIV care engagement, and death. Suicidal behaviour is criminalized in 20 countries worldwide, including Tanzania, where culturally appropriate interventions are lacking. We describe the experiences of counsellors who screened patients as the initial procedure in a randomized controlled clinical trial aimed to reduce suicide and depression, and improve HIV care engagement in Kilimanjaro, Tanzania. The clinical trial was registered at clinicaltrials.gov (ID: NCT04696861). We conducted in-depth interviews (IDIs) with 10 HIV counsellors and four mental health workers. Interviews were held 3 months post-enrollment of participants. Data was collected from March to August 2023. We referred to a brief screener developed for the trial, combining the PHQ-2 for depression and one question on suicidal ideation. IDIs focused on the frequency of depression and suicide assessments before and after the trial; the nature of assessments and referrals; perceived significance, acceptability, and feasibility of the screening process; and opinions on the criminalization of suicide. Data was analyzed using NVivo. Themes were identified, collected, compared, combined, and tabulated. Differences were resolved by the first three and final authors. Our findings revealed an increased focus on mental health assessments and referrals since the start of the trial, perceived high necessity of integration of mental health screening, and a high acceptability and feasibility of screening. Participants consistently reported increased mental health awareness and a positive overall experience of screening. Counsellors favoured abolishment of laws against suicide due to their hindering support-seeking. In a mental health resource-limited setting, these findings highlight the need for targeted and integrated non-specialist interventions. Feedback from counsellors indicated that screening was acceptable and feasible; further research is needed to assess the sustainability of screening.

## Background

Tanzania has an estimated 1.7-1.9 million people living with Human Immunodeficiency Virus (PLHIV) [1], the sixth largest population of PLHIV in any nation worldwide [2]. Depression and suicidal ideation are disproportionately more common among PLHIV in Tanzania [1,3,4], and deaths due to suicide occur more than twice as often among PLHIV in Tanzania than in the general population [5]. The World Health Organization (WHO) estimates over 3000 people die by suicide in Tanzania per year, which is the fourth most in Africa, and with sharply increasing rates [1,6]; studies further estimate that 26% of these deaths occur among PLHIV [7].

Resources for mental health care in Tanzania are critically lacking, with fewer than one psychologist or psychiatrist per million people [5,8]. Furthermore, routine screening for common mental health issues such as depression and suicidal ideation has not been implemented in most healthcare settings, including HIV care [1,4]. Previous literature highlights cross-effects between mental health and physical health even after adjusting for confounding factors [9]. Studies in the Kilimanjaro region of Tanzania have begun to elucidate factors associated with high rates of depression and suicide among PLHIV, including shorter duration since HIV diagnosis, female gender, being single, experiences of violence, and non-adherence to antiretroviral medications [4,10,11]. These challenges highlight the critical need for cost-effective and sustainable solutions such as task-shifting mental health care [4,12].

Barriers towards better addressing mental health challenges in this context include stigma, low awareness, and limited integration of mental health services into healthcare [1,5,13,14]. Research reveals that these shortcomings lead to inadequate or fragmented mental health care, and often attention is given to only the most severe conditions [12]. Furthermore, Tanzania is one of 20 countries worldwide where suicidal behaviour is criminalized [15], Section 217 of the Penal Code states: “Any person who attempts to kill himself is guilty of an offence” [16]. This may impact patients’ willingness to disclose suicidal thoughts to care providers and leads to many cases going unrecognized [17].

Nurses and other allied health professionals in Tanzania receive limited training in mental health [18] and may hesitate to implement routine screening or conduct mental health assessments due to low preparedness [19,20]. They may not recognize the significance of challenges [21,22], may be overburdened by their current responsibilities, or may fear exacerbating mental health challenges by talking about them [20,23]. In light of these obstacles, studies from Kilimanjaro have highlighted the feasibility of task-shifting, utilizing brief, nurse-led screening of depression and suicidal ideation paired with counselling that can be implemented and integrated into regular HIV care to plan for a patient’s safety and link the patient to a higher level of care as appropriate [5].

The IDEAS for Hope (Addressing Suicidal Ideation, Depression through HIV Education and Counselling, Advancing ART Adherence, and Reducing Stigma for Fostering Hope and Resilience among People Living with HIV) study in Kilimanjaro has implemented brief screening for depression and suicidal ideation in routine HIV care appointments, as well as a brief, telehealth-delivered counselling intervention to reduce depression and suicidal thoughts and behaviour and improve HIV care engagement [1]. The objective of this study was to conduct qualitative in-depth interviews (IDIs) with staff who participated in screening and referred patients for telehealth-based counselling, and with staff who received patient referrals for mental health support, to better understand their experiences and perceptions of these new procedures. We also aim to describe the landscape of mental health services offered within HIV care prior to this study.

## Methods

### Study design

The IDEAS for Hope clinical trial was registered at clinicaltrials.gov (ID: NCT04696861). New mental health screening, counselling, and referral strategies were implemented at four HIV clinics in the Kilimanjaro region [5].

### Study setting

The four research sites are all located in an urban setting in the Kilimanjaro region of Tanzania. Kilimanjaro Christian Medical Centre (KCMC) serves as the zonal referral level facility, with a catchment area of approximately 15 million people. KCMC receives patient referrals from the other three sites, for the most complex health challenges. KCMC also has the highest capacity for mental health specialty care in the Kilimanjaro region, overseeing mental health service provision at the other three facilities. Mawenzi Regional Referral Hospital (MRRH) is the designated tertiary-level facility for the Kilimanjaro region, receiving patient referrals from all six districts in the region, and is home to the only inpatient psychiatric unit in the Kilimanjaro region. Majengo and Pasua health centres are relatively smaller primary health care facilities that also provide HIV care. Majengo has one mental health professional, a Bachelor’s-level counsellor, and Pasua has none and relies on support provided by a social worker and external referrals for mental health treatment.

### Participant recruitment

Recruitment started on 6^th^ March 2023 and ended on 31^st^ August 2023. We conducted IDIs with 10 healthcare workers at the four HIV clinics which were involved in mental health screening and referral. We conducted additional IDIs with four mental health professionals responsible for standard-of-care mental health treatment at these sites, who received patient referrals after brief study-provided counselling. The selection of participants was purposive, considering the gender, rank, year of work experience and clinical roles of the healthcare providers. We decided on this number of IDIs based on the participants’ roles and having reached saturation across our data collection. The participants included HIV nursing counsellors, clinical officers (equivalent to a physician assistant), a psychiatrist, Bachelor’s-level clinical psychiatry practitioners, a mental health nurse, a health attendant, and a general practitioner.

### Intervention procedures

Screening consisted of the two-item version of the Patient Health Questionnaire (PHQ-2) and a single item assessing suicidal ideation S1 Fig. This PHQ-2 assesses anhedonia, i.e., how often in the past two weeks the patient has had “little interest or pleasure in doing things” and depressed mood, i.e., “feeling down, depressed, or hopeless” [24]. Items are responded to on a Likert-type scale from 0-Not at all to 3-Nearly every day for a total score of 0 to 6. This scale has been shown to have similar validity to the longer 9-item version of the PHQ while taking less time to administer [25]. For this study, we used the standard cutoff of scores 3 or higher indicating likely depression. Nurses screened for suicidal ideation using one yes/no item derived from the Columbia Suicide Symptom Rating Scale (C-SSRS) [26], “In the last month, have you had any actual thoughts of killing yourself?”.

Healthcare workers at the four HIV clinic sites were responsible for informing all patients of the new screening procedures, administering a brief screening instrument assessing depression and suicidal ideation, and referring patients who screened positive for depression and/or suicidal ideation to a research team counsellor for brief counselling. After brief counselling, all patients received referral information to continue with standard-of-care mental health treatment. Patients who screened positive for likely depression, suicidal ideation, or both were referred to a trained study counsellor who conducted additional assessment and provided brief counselling according to WHO’s Problem Management+ [27] and an adapted version of Stanley and Brown’s protocol for Safety Planning [28]. Study counsellors held either a Bachelor’s degree in psychology or a two-year Diploma in nursing. The positive experiences and feasibility of task-shifting of these counselling sessions are described elsewhere [29]. Patients with an active plan or intent to attempt suicide were directly accompanied to a mental health provider for further assessment and treatment.

### Data collection procedure

HIV nurses who conducted screening and mental health providers who received referrals at the study clinics were invited to complete a 20-to-55-minute semi-structured qualitative interview. Participants were approached at their clinic and invited to participate, and they all provided written informed consent before the interview. IDIs were guided by the first author, a psychiatrist and early career research fluent in both Swahili and English. IDIs were conducted in Swahili in a private study office at the clinic. Participants received compensation of 10,000 Tanzanian shillings (approximately $4.50 U.S.) or a similarly valued gift of thanks for their participation.

The IDI guide was developed and elaborated according to evidence-based guidelines [30]. Interviews began with the collection of brief demographic information, including the participant’s age, gender, and years of experience in HIV or mental health care. IDIs focused on the respondents’ experiences and actions during the mental health screening and referral process. The IDI guide included questions about the specific clinical context where they work, the types of mental health services offered within HIV care prior to this study, the reactions of the patients to screening and referral, and their interactions with the study counsellors and clinic staff during the study. Other questions explored subsequent administrative decisions related to the sustainability of screening and referral after study completion, as well as opinions on the criminalization of suicide in Tanzania. Topic guide questions were designed to cover all target areas, but IDIs were adapted to fit with the participant’s area of expertise/work. After the IDIs, interviewees were invited to ask questions or offer further relevant information that they felt might have been missed during the interview.

### Qualitative data analysis

IDIs were conducted in Kiswahili and audio recorded. Audio files were transcribed and translated into English by two research staff members with Bachelor’s degrees, a minimum of two years’ research experience, and written and verbal fluency in both English and Swahili. The transcripts were then transferred to NVivo 14 for analysis. The study team used thematic analysis; to gain familiarity with the data, KM, JM, MP, and BAK first read through the transcripts independently and highlighted meaningful segments of the raw text. They used an inductive process to identify, review, define, and name emergent themes and sub-themes. These themes were referred to as the co-author read through the transcripts again and used to find codes, and thereafter prepare a report [31]. BAK serving as the data auditor and guided the resolution of differences between interpretations by the first three authors until conclusions were reached.

### Ethical approval and consent to participate

Clearance to conduct this study was granted by research committees at Kilimanjaro Christian Medical Centre (Certificate No. 2523), Duke University Health System (Reference ID: Pro00107424-CR-3.2), and the Tanzanian National Institute for Medical Research (NIMR/HQ/R.8c/Vol.I/2420). Prior to IDIs, informants were approached for their written informed consent.

## Results

We report the findings of IDIs with 14 participants, exploring their experiences and perceptions of an innovative, newly integrated, and brief mental health screening in HIV care.

Below (Table 1) is an overview of the demographic information of the study participants.

**Table 1:** Participant characteristics (n=14).

Participant	Age (Mean = 39)	Gender	Role	Years of experience in HIV or mental health care (Mean: HIV care = 5.1, mental health care = 6.2)
1	48	Female	HIV nursing counsellor^*^	12
2	24	Female	General practitioner^*^	2
3	32	Male	Health attendant^*^	3
4	42	Female	HIV nursing counsellor, Nurse In-charge^*^	5
5	43	Female	HIV nursing counsellor^*^	5
6	45	Female	HIV nursing counsellor^*^	13
7	50	Female	HIV nursing counsellor^*^	3
8	44	Female	Mental health nurse#	11
9	33	Female	Clinical psychiatry practitioner#	2
10	27	Male	Clinical officer^*^	2
11	24	Female	Clinical officer^*^	1.5
12	59	Male	Medical officer#	10
13	40	Male	Clinical psychiatry practitioner#	5
14	33	Female	Psychiatrist#	3

* HIV care; #Mental health care.

### Mental Health Services Offered Within HIV Care in the Past

Before the study, a HIV clinic staff member reported that regular mental health screening was not conducted within HIV care:

*“Before the study, we never talked about things like that, to ask someone about suicide and how they feel, we never asked them.”*

(Participant #1)

Reasons for referral for mental health care included persistent non-adherence to HIV medication, psychological distress, disorganized speech, depression, and suicidal thoughts. As one participant mentioned,

*“When he comes here to the clinic you might find him at times talking a lot, things you don’t understand, or at times he might be crying. Or if you talk to him, he doesn’t speak, so from there, we will realize that there is a big problem. Perhaps he needs help, so that’s when we take him.”*

(Participant #7)

Before this study, HIV clinic nurses would discuss patients who were experiencing mental health challenges amongst the HIV care team and, when appropriate, would inform treatment supporters or family members of the patient about their concerns as a means of encouraging them to seek mental health treatment.

*“Yes there were [reasons for referral], there were some conflicts that usually arise within the families of our clients… so sometimes we would call for assistance from the psychologists but we would start with the social workers here at CTC.”*

(Participant #1)

Multiple HIV clinic nurses reported that before the study, they had little knowledge of depression or suicide and no experience in suicide risk assessment.

*“We did not know how to ask these questions”*

(Participant #6)

*“We see that there were some things that we were not doing… we were clueless”*

(Participant #3)

Mental health service providers reported that before the study they conducted frequent consultations on inpatients across other hospital units, many of whom were known to be living with HIV. Mental health workers were also sometimes consulted to de-escalate and resolve conflicts among couples or families in the hospital or clinic.

Before the study, multiple challenges hindered the seamless referral of patients to the HIV clinic to receive mental health care. Challenges included lack of available mental health providers, lack of financial support to pay for mental health care, distance/transportation to the clinic, and mental health providers’ overburdened workload. HIV providers also expressed concern about the time and effort required to add mental health screening, counselling, and referral to their current role given their high workload. These challenges contributed to a lack of assessment, referral, and delay in receiving mental health care, as well as difficulties with follow-up with the mental health clinic when a referral was made or mental health care was initiated.

*“Most of the times we wanted to escort the patient to the Mental Health Department, but other patients were waiting to receive services”*

(Participant #3)

Participants remarked that prior to the study, they were aware of the complexities of discussing suicide with patients, particularly because of the legal status and subsequent fear of disclosure, which contributed to a lack of suicide risk assessments.

*“When most patients have mental health problems, you find they and even their relatives will deny it [suicidal behaviour]. They hide it because they think that it is possible that he may enter the hands of the law”*

(Participant #10)

*“In the beginning we were not asking those questions”*

(Participant #5)

However, participants remarked that it was uncommon practice for providers to breach patient confidentiality, or report patients to legal authorities:

*“That’s more political, for us we were always looking at whether someone has a mental health problem. We know that is patient. We can defend in him in one way or another.”*

(Participant #9)

### Screening experiences

#### i. Patients.

As part of the current study, HIV clinic staff implemented universal screening for depression and suicidal ideation during routine HIV care appointments. HIV clinic staff reported that some patients expressed surprise and suspicion when being screened:

“*What questions are you asking me? I can’t think of hanging myself ever, don’t ask me*.”

(Participant #4)

However, when informed about the purpose of the screening and study, patients became less resistant and began to open up. Many patients expressed relief after the screening:

“*He begins to explain his things from beginning to end. It’s a good thing that he gets to talk openly and he’ll leave happy.*”

(Participant #6)

Other patients were reported to react positively, being understanding and excited that something new was happening at the clinic to support patients.

Some participants reported that patients attending their regular HIV clinic appointments were hesitant to be screened because they assumed it would take a long time. Reassurance that the screening process was brief and building a rapport with patients often helped them to agree to being screened.

*“There were clients that after being screened, and found that they fit the criteria, they tell you, “Right now I don’t have time and I have to go somewhere so I won’t be able to go there [for counselling]”.”*

(Participant #11)

*“You would explain the importance of which it takes a lot of time for them to understand”*

(Participant #6)

A few patients still refused to be screened in spite of the efforts of HIV clinic staff because they had urgent business to attend to outside the clinic.

#### ii. Staff.

While many of the HIV clinic staff reported smooth and positive implementation of the screening process, a few participants reported environmental and structural barriers to screening and referral. For example, one participant described occasional challenges related to privacy:

“*Due to the smallness of our buildings, you find that someone enters and has come to take something from the office because they are sharing the office*.”

(Participant #8)

Other staff reported limited paper and pens as a reason for intermittent screening.

*“We were using our own resources, something that is not so easy to sustain”*

(Participant #1)

### Perceptions

#### i. Perceptions of the Study.

Participants unanimously reported that the study was advantageous to the service users and health system, with noticeable improvements among patients care engagement.

*“Overall, it was a good exercise. Beneficial to patients, but even us health care providers.”*

(Participant #10)

#### ii. Perceptions of suicide.

The Penal Code of Tanzania prohibits attempting suicide and considers it a punishable offence. However, there were varying opinions on whether this law is helpful. Most participants felt that criminalizing suicide is misguided, as people who are struggling with suicidal thoughts require support and treatment, not punishment. Participants further remarked that these laws were a result of ignorance on mental health issues.

*“I would say it’s not a crime because someone has already had an impact on their mental health”*

(Participant #5)

*“It [the law] should be amended, and people should be free to go to the hospital to seek mental health care”*

(Participant #13)

*“Until someone reached the point of wanting to die by suicide… Instead of judging them, we should understand that this person must have passed through a lot of painful experiences.”*

(Participant #1)

Several participants expressed the belief that labelling suicide as a crime in Tanzania could discourage individuals from seeking help and make it difficult for health workers and people in the community to aid those in need. One participant said:

*“It has a big impact, for people not to come looking for support”*

(Participant #8)

*“The government should recognize that, people don’t ask for help because they will be judged”*

(Participant #8)

On the other hand, two participants believed that making suicide a criminal offence in Tanzania could potentially deter individuals from attempting or committing suicide and encourage them to seek out alternatives. One participant stated,

*“I believe that suicide being criminalized could potentially reduce the number of suicide cases in the country.”*

(Participant #2)

Several participants suggested that early identification of patients with mental distress, followed by offering help and support instead of reporting them to authorities, could prevent suicidal behaviour.

*“People should be screened and diagnosed early, get help before they get to attempt suicide.”*

(Participant #5)

### Importance of Mental Health Within the Context of HIV

Mental health staff within the clinics believed there was a causal link between HIV and mental health challenges, and that they saw a considerable number of PLHIV seeking mental health support. Mental health providers also noted that a large number of PLHIV who were experiencing emotional challenges were also experiencing challenges with adherence to HIV medication and treatment. One mental health nurse noted:

“*[If HIV] has affected him psychologically, we are often brought in to talk to them and consult with them, and also for HIV-positive patients we have been seeing a lot of them, especially those with poor adherence to medications and those who have lost hope.*”

(Participant #8)

### Impact of the Study

#### i. Impact on Care Provision and Patients.

Both mental health and HIV providers said that they had predominantly positive experiences with the study, which involved the implementation of universal screening and, for patients experiencing depressive symptoms or recent suicidal thoughts, referral to study staff for brief counselling and linkage to additional care as needed. The screening process was remarked as a new but welcomed procedure.

*“I can say there is a big change, because if you look at our clinic, every patient is now screened. It has improved the way we serve our clients”*

(Participant #8)

Providers noted the positive reactions of patients, who expressed gratitude for the opportunity to discuss their mental health concerns. Study staff were commended for their accessibility, effective communication, and appropriate support for both HIV clinic staff and patients, all of which contributed to a smooth study process.

*“You see the relief they [patients] have when they leave”*

(Participants #8)

*“We also had patients who refused to take their medications because of suicidal ideation, and after detecting that and referring them, they are now back to living a normal life and are continuing with their lives”*

(Participant #1)

*“Our sister [counsellor] who was involved, we thank her because whenever we called her or when we needed her, she would come”.*

(Participant #3)

Most of the participants in the study reported that the intervention had a positive impact on the mental health of the patients. They observed that patients were less resistant to sharing their struggles, more adherent to their medication regimens, and more open to receiving help. One participant remarked:

“*Those who wanted to hang themselves, those who gave up, even those who didn’t even want to take medicine…they’re okay now. Right now, they’re coming to the clinic peacefully*.”

(Participant #5)

#### ii. Impact of the Study on HIV Care Staff.

Staff within HIV care unanimously reported increased awareness of depression and suicide, were surprised by their prevalence, and felt more comfortable screening patients for these challenges.

*“We knew there were mental health issues but did not have a deep understanding of it. This research helped us a lot because these things now sit at the tips of our fingers”*

(Participant #10)

Additionally, staff felt the implementation of screening had normalized mental health challenges:

“*Because mental health challenges are also something that happens to staff, they have been motivated to do the screening*.”

(Participant #8)

As staff were reminded that depression and suicide exist in their communities outside of the hospital setting, they felt more empowered to discuss it:

“*One of the things I realized is it’s not just our patients. Even in society, these challenges are also on the streets where we live. When you have the opportunity…talk or educate people. I feel very free to talk about it.*”

(Participant #10)

#### iii. Implementation barriers.

The screening and referral process also introduced some challenges. One challenge was that some nurses and providers already felt overburdened in their existing roles, and this sometimes led to challenges in completing the study activities. For example, nurses had to dedicate time to the screening and referral for counselling, which sometimes required adjustments in their daily schedules to accommodate research activities. However, participants also commented that the challenges with time consumption decreased over time as they gained gexperience with screening.

*“It took a lot of time when we were not experienced, so it led to challenges with other activities but for someone who knew what they were doing it became easier so in the end, I don’t think time was wasted much.”*

(Participant #14)

Referrals from HIV care to mental health care also led to additional workload for the few mental health workers at the clinic, which sometimes contributed to longer wait times for existing patients or those seeking mental health care from other channels. At times, HIV clinic staff felt that the mental health workers were not responding appropriately to referrals. For example, one HIV provider noted:

*“We are all humans, and we can have some inconveniences. The counsellor allocated to work here is sometimes caught up with his other work, so he is not able to work here as effectively as we would like.”*

(Participant #1)

Juggling HIV nursing duties alongside research responsibilities was also cited as a source of strain. An HIV nurse reported:

*“You have to do the nursing work and you are also asked to do the work needed in the research. This truly did affect the working. Sometimes we even had to shorten the time used for morning reports so that we could have enough time for the research before the doctors come in to see the patients.”*

(Participant #1)

Ultimately, only two healthcare workers described challenges with the new process under study, while the others were quite positive about its impact on the clinics and patient care more broadly. The constructive feedback offers valuable insights for future screening and referrals, emphasizing the importance of adequate staffing and seamless integration of these activities into existing workflows.

#### iv. Sustainability.

Sustainability was a recurring theme in the interviews, with most participants stressing the need for the intervention to be sustainable in the long term. Several participants expressed hope for the program’s expansion across Tanzania to reach more patients in need of mental health support. Moreover, the research study was perceived as beneficial for both HIV providers and patients, with some expressing a strong desire to continue the intervention beyond the project’s duration.

*“We wish that this will go on because it has become an additional treatment… we were holding on to the medicine, but there are other things that are completely psychological”*

(Participant #3)

Following the intervention, participants noted an improvement in the attention given to mental health within the HIV clinic. Most participants cited an increase in referrals for mental health services for patients in the HIV clinic and a stronger partnership between the HIV clinic and the mental health department.

*“Until now, we talk to the patients to see if they have a psychological challenge”*

(Participant #5)

*“After the research ended, we decided to continue”*

(Participant #4)

*“We have been getting a lot of referrals and sometimes the HIV clinic have been calling me to see them there”*

(Participant #8)

Nonetheless, one participant expressed concern regarding their clinic’s lack of an in-house mental health professional. As one counsellor stated:

“*Right now, when we get a client* [with mental health care needs]*, we don’t even know where we are taking him/her.*”

(Participant #2)

Approximately half of the interviewees felt there would be advantages to using telehealth for mental health treatment, particularly given the shortage of mental health professionals available to provide in-person care. However, they also highlighted concerns that might impact the ability to provide telehealth treatment, including unstable electricity and internet connectivity.

Staff at three of the four sites reported that screening continued even after the study ended because of the perceived benefits. However, there was a reported lack of motivation among some members of staff:

“*Others looked at it like extra work, that it’s not part of their duties and it is just here to increase their workload.*”

(Participant #1)

At one clinic, where they do not have a mental health professional on-site, screening did not continue post-study. The staff reported concerns about managing patients in a mental health crisis when a professional is not available close by:

“*We were worried about what we will do with the client if we happen to find one that needs to be seen by a psychologist since our hospital setting does not have a psychiatrist or psychologist.*”

(Participant #2)

### Recommendations

Most of the participants expressed a desire to continue offering mental health support within HIV care due to the high perceived need of patients in the clinics and the perceived benefit of the study intervention. Other recommendations included providing training on mental health to HIV care providers, scaling up the intervention to reach more people and more clinics, and extending the intervention to community sites.

*“I think we may need a seminar or some kind of training… to be able to speak to a patient is such a way that he/she is comfortable enough to open up and to be able to counsel them before we send the client to the psychologist”*

(Participant #2)

Some participants underscored the importance of increased government involvement and funding for mental health initiatives such as this intervention and suggested the research team advocate with the government to this effect. They also urged policymakers to recognize the impact of mental well-being on overall societal functioning and productivity. As one participant quoted:

“*If you are not fit socially, physically, mentally, or spiritually, you can’t work. There is no healthy person in crisis who can work.*”

(Participant #9)

Participants expressed that the HIV clinics and hospitals operated in isolation, with little collaboration or communication across clinics. To address this challenge, several providers emphasized the importance of finding external support to facilitate collaboration across clinics to expand mental health interventions. Moreover, they highlighted the benefits of networking among providers for sharing resources and expertise.

Participants expressed the importance of increasing awareness through community outreach and health education, as these would be vital strategies to reduce community- and patient-level mental health stigma and promote mental health literacy. Community leaders, such as religious leaders, community groups, and local government were listed as vital partners in these efforts.

*“Local communities’ village chairpersons, they also have to know things like this. The people trust them so much and respect them so much, that they won’t do anything unless the chairperson says so”*

(Participant #9)

Participants felt additional steps could be taken at the hospital by engaging hospital leadership for approval and endorsement of mental health activities, education, and outreach. Education for current healthcare staff, including short-term training and access to online resources, was deemed essential to improve mental health literacy and service delivery. It was recommended that short on-the-job training or workshops would increase awareness, confidence, and competency in conducting screening and providing basic support in non-mental health settings. Additionally, formal training and hiring of more mental health providers was identified as a crucial need to bridge the gap between the demand and availability of mental health services.

*“There are still few places that offer mental health training programs”*

(Participant #2)

*“I think every district needs to have one such hospital where mental health professionals are available”*

(Participant #2)

One participant described a crucial need to expand research and treatment capacity to address emerging issues such as substance abuse and gender-based violence, which are commonly linked to mental health challenges. Additionally, participants recommended that within their institutions, task-sharing and task-shifting were viable solutions to increasing mental health capacity and reducing delays in treatment. One participant expressed concerns about how the term ‘research’ can cause discomfort to some staff and patients and recommended emphasis on reassurance that ethical standards would be maintained throughout the study period.

*“We should be careful with using the word research because although the response is great participants still have concerns about confidentiality”*

(Participant #3)

Other recommendations included creating and adapting more screening tools, integration of mental health services in non-mental health settings, and strengthening relationships between departments and between sites to facilitate communication and appropriate referral for different types of healthcare, including mental health.

## Discussion

Depression and suicidal thoughts and behaviour are common mental health challenges among PLHIV. Suicide rates are increasing rapidly in Tanzania [6], yet are still likely an underestimate due to various factors including the criminalization of suicidal behaviour. With critically lacking mental health resources in this setting, one potential solution is the development of interventions that integrate mental health services in non-mental health settings or use task-shifting and -sharing to improve mental health treatment capacity [5]. This study aimed to explore the experiences of staff who participated in the screening of depression and suicidal ideation in HIV care, referring patients for telehealth-based counselling, and receiving patient referrals for mental health support.

### Implications for clinical practice

This study demonstrated that providers believe it is feasible to screen PLHIV for mental health issues in HIV care, especially when mental health counsellors are available to receive referrals. HIV care providers noted an increased awareness of mental health conditions within the patient population. They emphasized the practicality of integrating mental health screening into routine care with minimal disruption due to a lack of resources. The challenges identified, notably the scarcity of mental health professionals and time constraints, similar to other studies’ findings [20,32,33], underscore the need for effective resource allocation in the face of limited resources [34]. Some studies have also reported on other challenges such as providers’ knowledge and skills [35–37], and inadequate training in use of mental health screening tools [33]. Implementing task sharing and leveraging telehealth have significantly enhanced access to mental health services while reducing costs related to transportation and potential treatment delays. Our findings highlight the critical importance of utilizing existing healthcare frameworks to integrate new research activities seamlessly.

### Implications for future research

Future studies should continue to explore whether brief screening and referral for counseling delivered by telehealth can be an effective strategy for improving mental health and HIV care engagement. Wider scale implementation in under-resourced areas and a longitudinal study design could provide deeper insights into the long-term feasibility and benefits of integrating mental health screenings for PLHIV. Understanding these dynamics will be crucial for refining the approach and scaling up the intervention effectively.

### Implications for policy

The high prevalence of depression and suicidal thoughts and behaviour among people affected by HIV, coupled with the limited resources for mental health, makes the integration of mental health services in HIV clinics an urgent need. The absence of routine screening for mental health issues necessitates policy interventions to support this service, ensuring that individuals can access care and help when they need it. This is particularly relevant given the indirect positive effects on physical health when mental health is given attention [9]. The study also highlights the effectiveness of task-sharing mental health care to non-specialized professionals to address the existing mental health care gap. Training nurses to provide screening, counselling, and referrals would promote early intervention [38].

The criminalization of suicide presents a significant barrier to individuals impacted by HIV seeking mental health support. This also reinforces the negative perception of mental health concerns, exacerbating the issue. Rather than punitive actions, policymakers should explore legal reforms and allocate resources, funding, and assistance towards promoting mental wellness. Additionally, community outreach initiatives [39] aimed at fostering mental well-being and training peer mental health educators within the HIV-affected community can play a crucial role in reducing the stigma associated with mental health.

### Study limitations

We acknowledge the possibility of responder bias, since the participants were aware of the ongoing research and may have felt influenced to respond more positively. Throughout the research process, we reassured participants about confidentiality and encouraged them to respond truthfully with no risk of repercussions.

## Conclusion

HIV and mental health care providers viewed the brief mental health screening as feasible, acceptable, and beneficial with only occasional interference to daily routine. Screening procedures were also reported as beneficial to the staff themselves, raising mental health awareness and improving HIV care engagement. The majority of participants argued against the criminalization of suicide.

It is important to note the large pool of knowledge surrounding the impact of mental health challenges on the lives of PLHIV, including loss of life. Adding to this previous literature, we emphasize the urgency of early recognition and intervention for such challenges, and investigating the sustainability of similar interventions.

Our findings further underscore the knowledge and evidence surrounding task-shifting and -sharing, cultural appropriateness, and validity of interventions addressing depression and suicide among PLHIV. Additionally, these findings highlight the acceptability and feasibility of conducting non-specialist-led interventions in non-mental health settings, despite limited prior awareness, preparedness and integration of such mechanisms.

Furthermore, our study is one of the first in Tanzania to explore perceptions of the criminalization of suicide in Tanzania.

## Supporting information

S1 Fig Brief Screening Tool for Depression and Suicidality. (TIFF)

S1 Checklist Inclusivity in global research. (PDF)

## Abbreviations

ART: Antiretroviral therapy
C-SSRS: Columbia suicide symptom rating scale
HIV: Human immunodeficiency virus
IDI: In-depth interviews
KCMC: Kilimanjaro christian medical centre
MRRH: Mawenzi regional referral hospital
PHQ: Patient health questionnaire
PLHIV: People living with human immunodeficiency Virus
WHO: World health organization.
