# Supplementary figures and images for: HIV and mental health provider experiences of implementing brief depression and suicide screening among people living with HIV in Tanzania: A qualitative study
Source: PLOS Ment Health. 2025 Mar 13;2(3):e0000268. doi: 10.1371/journal.pmen.0000268 (PMC12798201; doi:10.1371/journal.pmen.0000268)

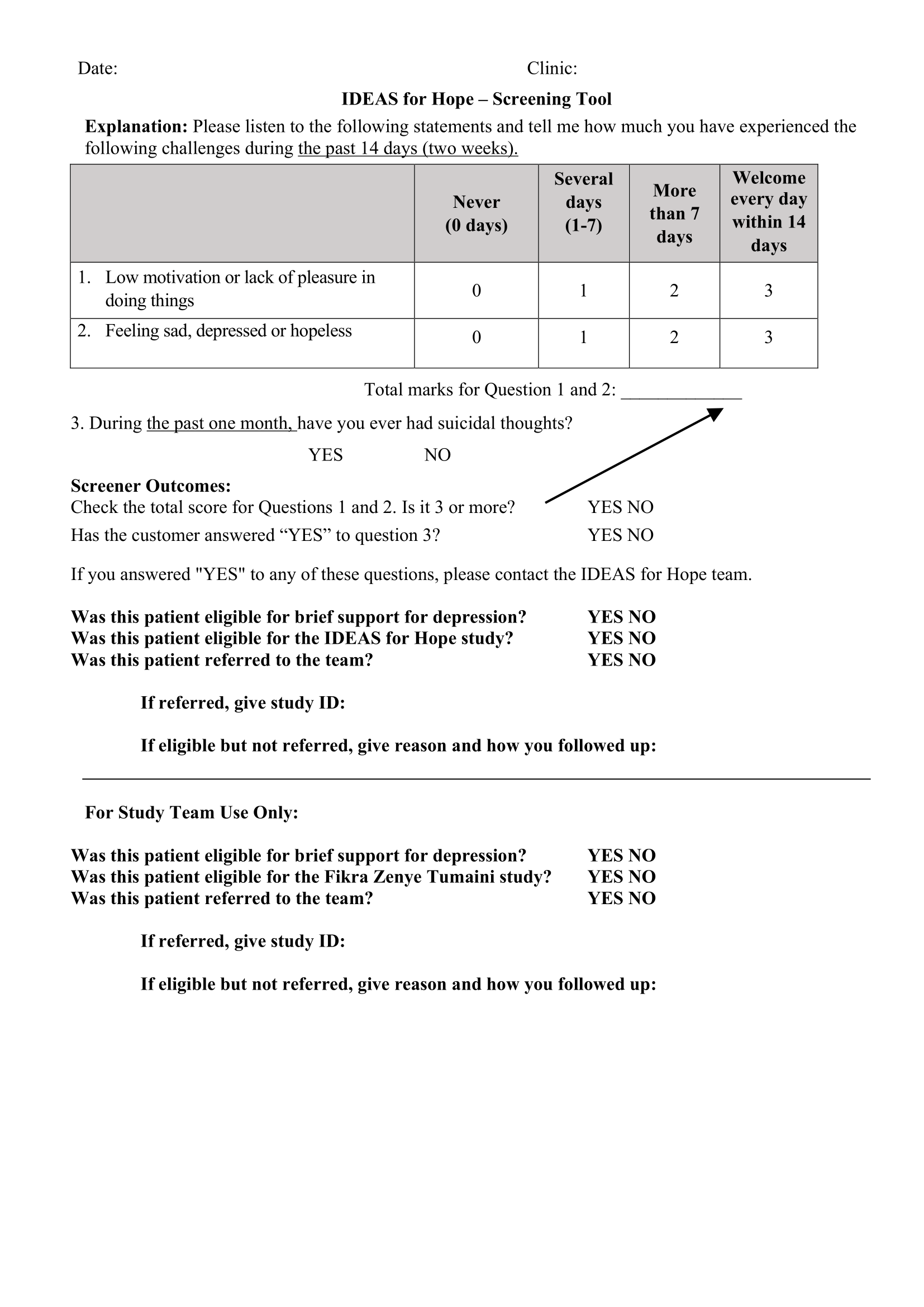

Supplement: S1 Fig — (TIFF) [file pmen.0000268.s001.tiff]
